# Reviewing research priorities in weed ecology, evolution and management: a horizon scan

**DOI:** 10.1111/wre.12304

**Published:** 2018-03-28

**Authors:** P Neve, J N Barney, Y Buckley, R D Cousens, S Graham, N R Jordan, A Lawton‐Rauh, M Liebman, M B Mesgaran, M Schut, J Shaw, J Storkey, B Baraibar, R S Baucom, M Chalak, D Z Childs, S Christensen, H Eizenberg, C Fernández‐Quintanilla, K French, M Harsch, S Heijting, L Harrison, D Loddo, M Macel, N Maczey, A Merotto, D Mortensen, J Necajeva, D A Peltzer, J Recasens, M Renton, M Riemens, M Sønderskov, M Williams

**Affiliations:** ^1^ Rothamsted Research Biointeractions & Crop Protection Department Harpenden Hertfordshire UK; ^2^ Department of Plant Pathology, Physiology and Weed Science Virginia Tech Blacksburg VA USA; ^3^ School of Natural Sciences, Zoology Trinity College Dublin Dublin Ireland; ^4^ Department of Plant Sciences University of California Davis CA USA; ^5^ School of Social Sciences The University of New South Wales Sydney NSW Australia; ^6^ Agronomy & Plant Genetics Department University of Minnesota St. Paul MN USA; ^7^ Department of Genetics and Biochemistry Clemson University Clemson SC USA; ^8^ Iowa State University Ames IA USA; ^9^ Knowledge, Technology and Innovation Group Wageningen University Wageningen the Netherlands; ^10^ International Institute of Tropical Agriculture (IITA) Kigali Rwanda; ^11^ School of Biological Sciences The University of Queensland Brisbane Qld Australia; ^12^ Plant Sciences Department Penn State University University Park PA USA; ^13^ Department of Ecology and Evolutionary Biology University of Michigan Ann Arbor MI USA; ^14^ School of Agricultural and Resource Economics Centre for Environmental Economics & Policy University of Western Australia Crawley WA Australia; ^15^ Department of Animal and Plant Sciences University of Sheffield Sheffield UK; ^16^ Department of Plant and Environmental Sciences University of Copenhagen Frederiksberg Denmark; ^17^ Department of Plant Pathology and Weed Research Newe Ya'ar Research Center Agricultural Research Organization (ARO) Ramat Yishay Israel; ^18^ Institute of Agricultural Science (ICA, CSIC) Madrid Spain; ^19^ School of Biological Sciences University of Wollongong Wollongong NSW Australia; ^20^ Department of Biology University of Washington Seattle WA USA; ^21^ Wageningen University and Research Lelystad the Netherlands; ^22^ Environment Department University of York York UK; ^23^ Institute of Agro‐environmental and forest Biology National Research Council (IBAF‐CNR) Legnaro Italy; ^24^ Molecular Interaction Ecology Radboud University Nijmegen Nijmegen the Netherlands; ^25^ CABI Surrey UK; ^26^ Graduate Group in Plant Science School of Agriculture Federal University of Rio Grande do Sul (UFRGS) Porto Alegre Brazil; ^27^ Department of Plant Physiology Faculty of Biology University of Latvia Riga Latvia; ^28^ Ecosystem Processes and Global Change Landcare Research Lincoln New Zealand; ^29^ Horticulture, Botany and Landscaping Department Agrotecnio, ETSEA Universitat de Lleida Lleida Spain; ^30^ Schools of Biological Sciences & Agriculture and Environment Australian Herbicide Resistance Initiative and Institute of Agriculture The University of Western Australia Crawley WA Australia; ^31^ Department of Agroecology Aarhus University Flakkebjerg Denmark; ^32^ Michael Williams & Associates Pty Ltd Natural resource Management Facilitators and Strategists Sydney NSW Australia; ^33^ Montana USA

**Keywords:** transdisciplinary research, integrated weed management, agroecology, weed adaptation, invasive plants

## Abstract

Weedy plants pose a major threat to food security, biodiversity, ecosystem services and consequently to human health and wellbeing. However, many currently used weed management approaches are increasingly unsustainable. To address this knowledge and practice gap, in June 2014, 35 weed and invasion ecologists, weed scientists, evolutionary biologists and social scientists convened a workshop to explore current and future perspectives and approaches in weed ecology and management. A horizon scanning exercise ranked a list of 124 pre‐submitted questions to identify a priority list of 30 questions. These questions are discussed under seven themed headings that represent areas for renewed and emerging focus for the disciplines of weed research and practice. The themed areas considered the need for transdisciplinarity, increased adoption of integrated weed management and agroecological approaches, better understanding of weed evolution, climate change, weed invasiveness and finally, disciplinary challenges for weed science. Almost all the challenges identified rested on the need for continued efforts to diversify and integrate agroecological, socio‐economic and technological approaches in weed management. These challenges are not newly conceived, though their continued prominence as research priorities highlights an ongoing intransigence that must be addressed through a more system‐oriented and transdisciplinary research agenda that seeks an embedded integration of public and private research approaches. This horizon scanning exercise thus set out the building blocks needed for future weed management research and practice; however, the challenge ahead is to identify effective ways in which sufficient research and implementation efforts can be directed towards these needs.

## Introduction

Weeds are defined here as any plants that have negative socio‐economic and/or environmental impacts, threaten global food security, biodiversity, ecosystem services and human health. Crop yield losses to weed competition have been estimated as 9% globally (Oerke, 2006), leading to estimates of annual economic losses of $27 billion and $3.2 billion, in the USA (Pimentel *et al*., 2005) and UK (Pimentel *et al*., 2001) respectively. In natural ecosystems, non‐native weeds have serious negative impacts on biodiversity and ecosystem functioning (Ehrenfeld, 2010; Simberloff *et al*., 2013). Invasive weeds may also result in serious consequences to human health through, for example, increased loads of allergenic pollen (Hamaoui‐Laguel *et al*., 2015). Impacts of weeds in current systems are likely to get worse rather than better, due to increased long‐distance trade, climate change, altered disturbance patterns, herbicide resistance and other factors, making improvements in weed management ever more urgent.

The global human population is projected to increase to 9 billion people by 2050, with conservative estimates suggesting an associated increase in food consumption and demand of 50% (Royal Society of London, 2009). This demand will need to be satisfied without increasing the global area of agricultural land, with fewer inputs and with a lower environmental impact, a concept described as ‘sustainable intensification’ (Royal Society of London, 2009; Tilman *et al*., 2011; Struik & Kuyper, 2017). For sustainable intensification to close the gap between theoretically attainable and realised crop yields (the ‘yield gap’, van Ittersum *et al*., 2013) whilst reducing negative environmental impacts, weed management strategies will require continued innovation, particularly considering the evolution of resistance to existing control measures (Godfray *et al*., 2010) and the continued introduction and spread of novel weeds or weedy traits (Driscoll *et al*., 2014). Climate and environmental change may also alter competitive interactions between agricultural weeds and crops, meaning that, over time, the nature and distribution of the most yield‐limiting weeds may change (Fuhrer, 2003). Additionally, the ecological impacts of invasive weeds are profound (Vilà *et al*., 2011) and are expected to worsen with global environmental change (Bradley *et al*., 2010). Existing management strategies for invasive plants are often proving ineffective at producing long‐term benefits (Pearson *et al*., 2016).

The converging challenges of global food security, climate change, environmental degradation, escalating rates of plant invasion, evolution of resistance to herbicides and the systemic failure to adopt integrated weed management (IWM) pose a stark challenge to the fields of weed ecology and management. Current trends suggest that weed problems will worsen in the next 10–20 years, becoming an even more intractable barrier in efforts towards the sustainable intensification of agricultural production and the preservation of natural habitats. It is critical that future efforts be more coordinated, collaborative, innovative and conducive to adoption. These challenges provide a timely opportunity to readdress the question ‘what are the future research priorities in weed ecology and management?’.

In June 2014, a group of 35 scientists engaged in various aspects of weed research and practice, spanning agricultural and invasive weeds, genetics and evolutionary biology, ecology, weed management and social science assembled at a workshop in Benasque, Spain, to consider future dimensions in weed biology and management. To facilitate those discussions, a horizon scanning exercise was performed (Sutherland *et al*., 2006; Grierson *et al*., 2011; Ricciardi *et al*., 2017). Before the workshop, invitees were asked to submit three to five ‘key questions’ that they considered to be major challenges for the discipline of weed ecology, evolution and management in agricultural and invaded natural systems over the next five to ten years. Through individual reflection and facilitated group discussion, the 124 questions submitted were ranked in importance. The top 30 ranked questions are presented here (Table 1) and form the basis of the commentary that follows. A full list of the submitted questions is included as supporting information, together with further details of the ranking exercise.

**Table 1:** The 30 top‐ranked current and future research questions in weed ecology and management. Questions are grouped and discussed under seven research themes

Rank	Question	Theme
1.	How can weed ecologists engage with society, government and private enterprise to facilitate multi‐stakeholder efforts to manage weedy and invasive plants?	Transdisciplinary research
2.	How can we work with social scientists to best co‐ordinate weed prevention and control efforts amongst multiple stakeholders?	Transdisciplinary research
3.	What is the role of epigenetics in weed plasticity and adaptation in agroecosystems?	Weed evolution
4.	How will natural species dispersal in response to climate change affect our definitions of invasive plant species and our tolerance of them?	Climate change
5.	How important is weed functional diversity in maintaining ecosystem function and reducing crop yield loss from weed competition?	Agroecology
6.	What is hampering the adoption of integrated weed management strategies? What are farmers trying to tell us?	Adoption
7.	How do we increase productivity and species diversity in the arable land at the same time?	Agroecology
8.	Can we predict which species will become more weedy/invasive with global warming?	Climate change
9.	What is the role of plasticity vs genetic variation (neutral/adaptive) in aiding/hindering adaptation and survival of weedy species?	Weed evolution
10.	What role does the soil microbiome play in regulating weed populations and their response to management?	Agroecology
11.	How can farming systems be designed for greater resilience to weeds?	Agroecology
12.	Can more heterogeneous cropping and weed management landscapes slow evolution of herbicide resistance?	Weed evolution
13.	Beyond the enemy release hypothesis, what is the role of biotic interactions in facilitating or hindering invasion rates?	Invasiveness
14.	A noticeable narrowing in content has occurred (in North America at least) within the ‘Weed Science’ community over the past decade, how do we move to broaden that scope?	Weed science
15.	Up to now weed management has been conducted primarily at the field level with a time horizon of a few months. What specific improvements can be obtained by using other spatial scales and time horizons?	a
16.	Will ecosystems experiencing disruption due to climate change be more invasible?	Climate change
17.	What ecosystem services arise from weeds in and near agricultural fields?	Agroecology
18.	How will climate change impact the distribution and competitive ability of weeds?	Climate change
19.	How do political/economic changes affect weed invasion? Can it be predicted or prevented?	Transdisciplinary research
20.	How does weed dispersal and management relate to characteristics of the associated social systems?	Transdisciplinary research
21.	How can farmer behaviour be best influenced to improve sustainability of weed management?	Adoption
22.	Weed problems are embedded in interactions across different levels. How do we account for interactions at plant, plot, farm, community, regional and national levels?	Agroecology
23.	Are there a set of functional traits that can predict the ecological impact of invasive plants?	Invasiveness
24.	How do we connect fundamental and applied research in weed research?	Weed science
25.	How can we attract excellent scholars into the field?	Weed science
26.	Are there some plant traits that we can be confident will be influenced by climatic change?	Climate change
27.	Does adaptation of invasive species in their introduced range reflect directional selection in the new range?	Invasiveness
28.	What factors do managers consider most important when choosing what and how to manage weeds/invasive plants?	a
29.	How can our research community avoid falling in the gap between ‘applied’ and ‘basic, hypothesis‐driven’ research funding programs?	Weed science
30.	Will weeds evolve resistance to non‐chemical control methods just as fast as to herbicides?	Weed evolution

a Note that questions ranked 15 and 28 were not categorised within a discrete research theme.

## Horizon scanning priorities and opportunities in weed ecology and management

In summarising the top‐ranked research questions (Table 1), seven salient themes were identified, each of which is discussed below.

### Transdisciplinary research

The two top*‐*ranked questions (and two others) placed a strong emphasis on the need for broadening research horizons, such that multistakeholder approaches to tackle weed problems and their management are fostered. Within these transdisciplinary frameworks (Lang *et al*., 2012; Jordan *et al*., 2016), weed ecologists, weed scientists, land managers, farmers, economists and social scientists should work together with agricultural, industrial and governmental stakeholders with an interest in tackling intractable weed problems (Graham, 2013; Ervin & Jussaume, 2014). Narrow framing of weed problems is less likely to engage the full range of stakeholders needed to devise and implement innovative solutions, and weed research must be considered in the context of wider efforts towards the design of sustainable farming systems. Continued technological innovation will be a key requirement for developing, testing and promoting sustainable weed management strategies, though a better balance is required between public and private sector research, development and funding for weed science. Whereas the public sector has been more inclined to focus on a range of systems‐based approaches, the private sector has continued to seek to develop ‘patentable’, technological solutions. Transdisciplinary science can serve to facilitate public–private partnerships that ensure that the most promising technological advances are deployed in systems that preserve their efficacy, maintain weed management and agroecosystem diversity and limit the potential undesirable environmental impacts of weed management.

### Adoption of integrated weed management

Two questions (ranked 6 and 21) identified the importance of continued efforts to increase, understand and incentivise adoption of IWM approaches (see Liebman *et al*., 2016). Underlying reasons for this lack of adoption are multifaceted and likely reflect a continued desire for ‘simple’ technological solutions, short‐term planning horizons and a failure by researchers to demonstrate and communicate the benefits of more integrated approaches. In part, future research approaches can address these questions using transdisciplinary frameworks that enable codevelopment of weed control technology and IWM systems, socio‐economic approaches to better understand farmer decision‐making and a wider framing of weed management challenges and solutions, including through public–private collaborations.

### Weeds as agroecological actors

A series of questions (ranked 5, 7, 10, 11, 17, 22) recognised the need for a greater research effort to reconcile the negative and positive impacts of weeds in agroecosystems. The interactions of weeds with other trophic levels and in relation to soil health and functioning can be important for delivering ecosystem services (Marshall *et al*., 2003). These services can include the provision of food, shelter and habitat for natural enemies of crop pests or for pollinating insects, the maintenance of vegetation cover during non‐cropped phases of the rotation to control soil erosion and for the enhancement of soil structure and function (Navas, 2012). As such, weed functional diversity may play an important role in enhancing crop productivity by reducing losses due to insect pests and maintaining or enhancing soil health. Trophic interactions may also play important roles in regulating weed populations through, for example, weed seed predation (Westerman *et al*., 2005; Franke *et al*., 2009) and microbial degradation of viable seeds in the soil seedbank (Chee‐Sanford *et al*., 2006; Müller‐Stöver *et al*., 2016). Of course, weeds may also increase the negative impacts of other crop pests by acting as hosts, shelter and/or food sources for plant pathogens (Wisler & Norris, 2005) and herbivores. Understanding biotic interactions between weeds and organisms at other trophic levels will be important for designing weed management strategies that enhance the natural capacity for ecosystems to regulate weed and pest populations. In this way, weed management strategies must be considered in the context of multifunctional landscapes that optimise crop production and environmental integrity whilst maintaining provisioning, sustaining and cultural ecosystem services. More diverse weed floras, selected for by more diverse weed management and cropping systems, may buffer systems against dominance by one or a few aggressive, resistance‐prone species, therefore increasing systemic resilience to weeds. Indeed, evidence from the long‐term Broadbalk experiment at Rothamsted Research has identified a negative correlation between weed diversity and crop yield loss (Moss *et al*., 2004). This observation suggests that increased weed diversity may not always have a negative impact on crop yield.

### Weed evolution

Workshop participants recognised a need to better understand the nature and importance of weed adaptation that underpins the evolution of weedy traits in agricultural and invaded natural systems (ranked 3, 9, 12 and 30). We are reminded of the words of Harper (1956) that ‘Arable weeds constitute an ecological group selected and maintained in association by their fitness for existence under conditions of crop cultivation. They comprise species that have been selected by the very practices that were originally designed to suppress them’. The ability of weedy plants to rapidly adapt to novel environments and anthropogenic management has been proposed as a key facet of the ‘weed syndrome’ (Vigueira *et al*., 2012). In agricultural systems, weed management, particularly the use of herbicides, exerts extreme selection pressure, and the capacity for weeds to rapidly evolve resistance to herbicides has been demonstrated extensively (Powles & Yu, 2010). Further, one of our questions acknowledged the need to also understand adaptive potential in relation to cultural weed management. In invasion ecology (see below), it is suggested that the success of invasive plants may be due, at least in part, to their ability to rapidly adapt to novel environments (Prentis *et al*., 2008). In the light of these phenomena, it has been proposed that weedy plants provide excellent model systems for studying contemporary adaptation in plants (Baucom & Holt, 2009; Neve *et al*., 2009; Vigueira *et al*., 2012). The extent to which phenotypic plasticity versus genetic variation is implicated in this adaptive potential is also an open question and, added to this, there is increasing interest in the role of epigenetic regulation in rapid evolution in plants (Becker & Weigel, 2012). In practical terms, answering these questions will be important for understanding how weed populations and communities respond to management strategies that aim to disrupt contemporary evolution through the design of heterogeneous landscapes, crop rotations and through the optimisation and adoption of IWM strategies.

### Invasiveness

Important questions relating to a better understanding of weed invasiveness (ranked 13, 23, 27), drew on themes developed in the two preceding sections. To what extent are invasions facilitated (or hindered) by interactions (or lack of) across trophic levels? What is the importance of post‐invasion evolution to invasion success? Invasion of an ecosystem by one species may be facilitated by native species or by previous invaders with sequential, facilitated invasions potentially leading to ‘invasional meltdown’ (Simberloff & von Holle, 1999). The success of invading species may be due to release from natural enemies, present in their native habitat, but absent in the invaded range (Williamson, 1996; Mitchell & Power, 2003), though reports of pathogen accumulation and subsequent population decline of invasive plant species after initial establishment have also been noted (Flory & Clay, 2013). Interactions between plants and soil microbes can also contribute to invasiveness (Klironomos, 2002; Callaway *et al*., 2004). Likewise, the failure of some species to invade may be due to the absence of mutualistic organisms in environments into which they are introduced (Richardson *et al*., 2000).

### Climate change

Global climate change (ranked 4, 8, 16, 18, 26) will impact the dispersal of weedy plants, the invasibility of agricultural and natural habitats and competitive interactions. Climate change is clearly recognised as a major driver for increased rates of plant invasion (Diez *et al*., 2012), and in agricultural situations, the geographical range over which weeds are highly competitive versus crops (the ‘damage niche’) may shift in response to altered cultivation practices associated with climate change (McDonald *et al*., 2009; Stratonovitch *et al*., 2012). The ability to better predict the introduction pathways and invasive potential of plants under climate change is critically important, so that those species likely to have the greatest negative environmental and socio‐economic impacts can be identified and anticipated. The ability to predict those plant traits that will be most impacted by climate change will help to understand which species will become more invasive under climate change. However, it is also important to recognise that a changing climate may result in wider ecosystem change and, in this context, the concept of what defines ‘native’ and ‘invasive’ species may also change (Webber & Scott, 2012).

### Weed science

A final set of questions (ranked 14, 24, 25, 29) raised several important issues relating to the future scope, definition, ambitions and approaches for the discipline of weed science (biology, ecology, management). A narrowing of focus was highlighted, invoking arguments about a ‘critical juncture’ for the discipline (Mortensen *et al*., 2012) and acknowledging that the advent and unprecedented adoption of herbicides for weed management have resulted in a discipline that has approached weed science from an increasingly narrow plant physiological versus a broader plant ecological perspective (Neve *et al*., 2014). Two questions addressed a similar issue about the need for our discipline to find a better balance between ‘applied’ and ‘fundamental’ science, and there was a consensus that much weed research ‘fell between the cracks’ in this regard. This may reflect a general perception that the study of weeds, even when focused on fundamental questions of weed biology, is an overtly ‘applied’ science, sometimes limiting access to more basic science funding. This ‘problem’ is less evident in plant invasion biology where scientific questions are successfully framed in the wider context of community assembly and ecosystem functioning and where the study of plant invasions is recognised as a means to address fundamental questions in plant ecology. In the future, the discipline of agricultural weed science should recognise and rise to the challenge of framing fundamental questions in plant ecology and evolution around the study of weeds in agroecosystems. Presenting weed science in transdisciplinary terms will similarly open up opportunities for those focused on the biology and management of weeds to expand the scope and focus of the discipline. These endeavours will facilitate wider efforts to attract the best scholars into the weed science discipline, with associated benefits in terms of raising the profile of the discipline, conducting fundamental science with ‘impact’ and addressing many of the challenges and opportunities highlighted by this horizon scanning exercise.

## Discussion

The overarching question that we have sought to address is how can we achieve weed management that is effective, economical, minimises negative environmental consequences and is robust to weed adaptation and future environmental change? From the preceding discussion, a single, unifying ‘meta‐theme’ has emerged: the need for more‐diversified agroecosystems to tackle intractable weed problems in ways that are economically and environmentally sustainable. Indeed, we observe that most of the research themes outlined above are pertinent to diversified agroecosystems and are largely of uncertain relevance in low‐diversity agroecosystems. The severe problems of weed management in low‐diversity systems are clear, and we call for a shift to focusing on critical scientific questions about weed management in more‐diversified systems. This effort will add impetus to wider efforts to enhance diversification in agriculture, which remains highly challenging in the face of many factors that favour more simplified cropping systems, production technologies and market drivers, even though such simplified systems now show limited sustainability.

*Transdisciplinary* approaches (Jordan *et al*., 2016) acknowledge the social, economic and political dimensions of weed management, engaging multiple stakeholders in the cocreation and codesign of IWM systems, overcoming potential barriers to subsequent *adoption* (Llewellyn, 2007; Wilson *et al*., 2009; Liebman *et al*., 2016) and ensuring a closer integration between public and private sector perspectives and drivers in weed management. More system‐based approaches to weed management can help to address some of the tensions and trade‐offs between economic, environmental and societal objectives, recognising the need for a closer integration between ‘technological‐’ and ‘*agroecological*’‐based solutions (Jordan & Davis, 2015). In this sense, we see opportunity and potential in drawing parallels with global healthcare challenges. Indeed, the concept of ‘one health’ in human and animal healthcare demonstrates an emerging consensus for a more holistic approach (Hueston *et al*., 2013) that recognises a strong environmental component and ecological interactions in the epidemiology of human and animal disease.

A more systemic, diversity‐oriented focus acknowledges that weeds can perform positive as well as negative roles in agroecosystems (Marshall *et al*., 2003; Navas, 2012), interacting with species at other trophic levels to deliver provisioning and regulating ecosystem services. Similar arguments can apply in natural systems invaded by non‐native weedy plants where there needs to be a clearer focus on those species which have the greatest ecological impact, accepting that some invasive species have few long‐term negative impacts. It is critical to recognise that these agroecological approaches do not envision cropping systems that tolerate large populations of competitive weeds. Instead, we argue that more diverse management systems that support and maintain a higher level of weed diversity will select against one or a few dominant, competitive species that typically come to dominate low‐diversity management systems. Whilst the notion of tolerating a more diverse weed flora may remain anathema to many, we point to the extensive evidence that current technological approaches have, with few exceptions, led to the dominance of one or a few, highly competitive, herbicide resistance‐prone species (see Délye *et al*., 2010; Ward *et al*., 2013; Owen *et al*., 2014). The move towards more‐diversified weed management is wholly consistent with the need to better understand and manage *weed evolution*. Low‐diversity weed management systems with heavy reliance on herbicides and without sufficient crop rotation impose strong directional selection for weedy traits, and a central tenet of IWM must be to diversify selection pressures to avoid the dominance of agricultural fields by one or a few highly adapted species, whether they be native or invasive in origin.

Global and regional *climate change* will continue to drive changes in plant species distributions and competitiveness, likely increasing the *invasiveness* of some species (Dukes & Mooney, 1999) and leading to new weed problems in agricultural and natural ecosystems. These challenges similarly call for broadening horizons in weed management to better understand the ecological and evolutionary drivers of invasion under climate change. Designing weed management systems that are more resilient to future invasions requires a similar focus on transdisciplinarity that acknowledges the social, economic and political dimensions of weed problems and the need for systemic ecological approaches that limit the invasion and ongoing adaptation of new weed species. As a direct outcome of our Spanish workshop, we organised a follow‐up meeting on transdisciplinarity in weed research in Canada in 2016. For this, we brought in a much wider range of disciplines and participants, including social scientists, extension scientists and local landowners. This workshop focused on establishing a common language and approach to integration of social and weed science to achieve the goals of effective long‐term weed solutions.

These challenges and their underlying research and philosophical questions present an opportunity for reinvention in *weed/invasion science* to broaden the scope of the discipline and, in doing so, to address emerging concerns about a disconnection between ‘basic’ and ‘applied’ science and the need to continue to attract the best scholars into the discipline. There is a healthy, ongoing debate about the future of the weed science discipline (Mortensen *et al*., 2012; Ward *et al*., 2014; Barrett *et al*., 2017; Harker *et al*., 2017). We should embrace that debate, avoiding fractious divisions that threaten to promulgate a false dichotomy between ‘technological’ and ‘agroecological’ approaches to weed management. The design of sustainable weed management systems that are robust to weed adaptation, weed invasion and future climate change and that place weed science in a broader context of sustainable intensification requires system‐based approaches that integrate technological and agroecological principles in diversified agroecosystems.

## Supporting information


**Data S1.** Materials and methods.
**Table S1.** The 124 pre‐submitted research questions that address fundamental and applied issues in weed ecology, evolution and management
