# Supplementary material for: Reviewing research priorities in weed ecology, evolution and management: a horizon scan
Source: Weed Res. 2018 Mar 28;58(4):250–8. doi: 10.1111/wre.12304 (PMC6055875; doi:10.1111/wre.12304)
Supplement: Supplementary file 1 — Data S1. Materials and methods. Table S1. The 124 pre‐submitted research questions that address fundamental and applied issues in weed ecology, evolution and management [file WRE-58-250-s001.docx]

**Supporting information**

**Materials and Methods**

*Workshop participants*

The horizon-scanning exercise was conducted as part of an international workshop (Benasque, Spain, June 2014), organised by the ‘ANdiNA’ group of weed and invasive plant scientists. Attendance at the workshop was via written application. Workshop attendees were selected to ensure an international coverage of scientists with diverse interests and expertise in weed ecology, evolution and management, spanning a variety of academic disciplines. Thirty-five scientists attended the meeting, including a professional facilitator specialising in natural resource management. There were 23 male and 12 female participants. Of the 34 specialists at the meeting, 15 only conducted research related to weeds, whilst 19 studied weeds as part of a wider research portfolio. Fourteen scientists focused solely on arable production systems, 11 on unmanaged systems and nine scientists had interests in both systems. Seventeen people identified themselves as weed ecologists, six as weed scientists, five as agricultural scientists, three as evolutionary biologists and three as social scientists. There were 14 early-career researchers, 11 mid-career and 10 established researchers. Participants were from 12 countries, spanning five continents. The majority of research was being conducted in Europe (20 participants), followed by North America (12) and Australia (11); there were fewer than five people carrying out research in each of South America, Asia, Africa and Antarctica. The majority of researchers were funded by government sources (78% of total funding), with smaller proportions of research funding arising from, industry (9%), non-governmental organisations (3%) and other sources (10%).

*Soliciting research questions.*

Before the workshop, invitees were asked to submit three to five ‘key questions’ that they considered to be major challenges for the discipline of weed ecology, evolution and management over the next five to ten years. It was suggested that these questions should address fundamental and applied questions that were broad in scope and integrative, rather than focused on specific problems in a subset of agricultural or unmanaged systems. In total, 124 questions were submitted (Table S1). Responses were collated and, based on a preliminary analysis, assigned to one of seven categories: ecology (36 questions); weed management (32); weed evolution (23); socio-economics and policy (13); weed science (general) (11) and climate change (9).

*Ranking of questions.*

All 124 questions were displayed on index cards under the seven category headings at the workshop venue. Each participant was requested to identify five gold (50 points), five silver (10 points) and five bronze (5 points) rated questions, to reflect their perceptions of important research priorities. Participants were requested not to vote for their own questions and to consider selecting questions from across a range of categories. A total score for each question was calculated. The top 30 ranked questions were identified and a subsequent facilitated discussion sought to converge and distil these questions into the themed areas discussed in the main text.

**Table S1.** The 124 pre-submitted research questions that address fundamental and applied issues in weed ecology, evolution and management

| **Submitted question** |
| --- |
| **Category: Climate change** |
| Are there some plant traits that we can be confident will be influenced by climatic change? |
| How will weed management in annual production systems be affected by changes in the frequency and intensity of rainfall events predicted by climate-change models? |
| Do new weeds/invasive species arise due to changing climate? Can it be predicted which species will become more weedy/invasive with global warming? |
| What are the main weed ecology issues to be addressed considering the effects of global change relating to the expansion of agriculture into deserts, savannas and natural forestry areas? |
| How does global warming affect the host-range and distribution of parasitic plants? |
| What synergisms exist between invasion and climate change biology? |
| How will climate change impact the distribution and competitive ability of weeds? |
| How will natural species movements or population translocations in response to climate change affect our definitions of invasive plant species and our tolerance of them? |
| Will ecosystems experiencing disruption due to climate change be more invasible? |
| **Category: Weed ecology** |
| Can invasion of invasive species be facilitated or inhibited by co-invaders or earlier/later arriving species? |
| Can global invaders be endangered species in their native range? Does this mean the search for 'invasiveness' is meaningless? |
| How can we more systematically document the impacts of plant invasions, in a manner that’s comparable across spatial, temporal and functional scales? |
| Which are the potential benefits of protecting weeds? How can farmers be compensated for protecting the ecological services provided by those plants? |
| Looking at the beginning of the last century we identify several theories of ecology, plant ecology and weed ecology that unify the knowledge and point out the future for new studies. Where are the new weed ecology theories based on recent science? |
| The high-productivity systems in the tropical areas are growing very fast. What are the key weed ecology issues to be studied in the new high-input agricultural systems in tropical areas? |
| Current studies have identified long-term crop management effects on weed population dynamics?  What are the strategies to identify these effects? |
| What is the ideal relationship of weed ecology with the “OMICs” technologies? |
| What is the role of the soil sub-surface medium in weed research? |
| Are there threshold impacts as environmental weeds increase at regional scales? |
| Biocontrol agents: What works in the long term and how well is it controlling spread of the weed? |
| Seed bank longevity and understanding losses from the system |
| Are there a set of functional traits that can predict the ecological impact of invasive plants? |
| When do invasive plants matter? In other words, how long or abundant before their ecological impact is meaningful? |
| At what point do invaders change an ecosystem past a tipping point? |
| How do changes in resource and disturbance regimes (herbicide, tilling, or climate change *eg* fire, precipitation patterns) affect the successful invasion of weeds and the long-term effect on limiting native persistence? |
| How could weed seed predation be promoted and supported in field crops to become a reliable and effective tool for IWM strategies? |
| How could the beneficial effect of weed presence for supporting biological pest control (nesting, shelter, food source) in field crops be maximized without causing excessive crop-weed competition? |
| How could crop-weed interactions be studied to identify possible positive effects of weed presence on crop growth in relation to nutrient cycling, nutrient uptake or soil pathogen control? |
| Beyond the enemy release hypothesis, what is the role of biotic interactions in facilitating or hindering invasion rates? |
| Which of the currently casual or naturalised weeds are the high impact invasives of tomorrow? |
| How do we increase productivity and species diversity on arable land at the same time? |
| Are there any group of weed species with similar traits, which are more important to preserve than others in the agricultural ecosystem? |
| Do we need fundamental research on weed ecology if robots will do the job? |
| Can we enhance weed seed decay by managing soil biota and/or soil structure? |
| Why is weed seed predation so variable across agroecosystems? |
| Can we develop quick methods or technology to readily assess weed seed banks in a field? |
| Is "weed" an artificial term for plants that are unwanted in the agroecosystem, or are there distinct traits, most probably, suites of traits, that make a species a potential weed or invasive plant? |
| How important are species, grouped as "weedy", for the ecosystems, and is weed diversity really important for pollinators and other organisms? |
| How important is weed functional diversity in maintaining ecosystem function and reducing crop yield loss from weed competition? |
| What role for DNA barcoding in weed science? |
| Will management for ecosystem service provision be in conflict with current views of invasive plants and their management? |
| What role does the soil microbiome play in regulating weed populations and their response to management? |
| When can management of landscape-scale patterns and processes provide new means of weed management? |
| What ecosystem services arise from weeds in and near agricultural fields? |
| Are weeds passengers or drivers of ecosystem change? |
| **Category: Weed evolution** |
| What are the invader driven adaptive changes/responses in communities that are experiencing new biotic selective agents? |
| Does apparent adaptation of invasive species in their introduced range reflect directional selection in the new range or does it result from genetic bottlenecks and genetic drift during the invasion process? |
| Do we know much about the scale of hybridization of invasive plants with native plants *in situ?* |
| Can integration of different levels of biological organisation (genomics, transcriptomics, metabolomics and ecology) give a better/different insight into what makes a species invasive? |
| Do particular evolutionary changes lead to greater invasiveness? Which genetic processes (e.g. genetic bottlenecks, admixture) play a role? |
| Is variation in metabolic (multiple) herbicide resistance the result of many loci of small effect or a few loci of large effect? |
| What is the dimensionality of the genetic basis of metabolic herbicide resistance with respect to the selective environment (e.g. different herbicides)? |
| Does the metabolic herbicide resistance ‘G’ matrix (with respect to different herbicides) look similar across different species? |
| How does the environmental context influence the evolution of multiple herbicide resistance (is GxE important)? For example, does environmental stress / climate variation impact multiple herbicide resistance? |
| Which regions of the genome are involved in adaptation to herbicide usage? Are the same regions involved every time multiple herbicides resistance evolves, or can different regions come into play in different contexts? |
| What are the ecologically important traits underpinning multiple herbicide resistance for which significant levels of standing genetic variation exist? |
| How important is post-invasion evolution in invasive plant species? |
| What is the role of epigenetics in herbicide resistance? |
| What is the role of plasticity vs genetic variation (neutral/adaptive) in aiding/hindering adaptation and survival of weedy species? |
| What is the efficacy of heterogeneous management strategies on the spread of herbicide resistance across the landscape? |
| How would the wide adoption of IWM strategies affect the evolution of weed communities in field crops in a mid- and long-term perspective? |
| Does space matter when understanding and predicting the evolution of resistance to herbicides? |
| Will weeds evolve resistance to non-chemical control methods just as fast as to herbicides? |
| To what extent is selection/adaptation important in plant invasions? |
| Does evolution in every instance of plant invasion (eg into different continents) follow a similar trajectory?  If not, how do these trajectories differ? |
| What is the role of epigenetics in weed plasticity and adaptation in agroecosystems? |
| Is stacking herbicide resistant traits in GM crops really going to delay evolution of herbicide resistance? |
| Where is the focus of the divergent evolutionary strategy (harmfulness vs endangered) of weed species? |
| **Category: Weed management** |
| Can we synthesize organic and herbicide-based weed management systems to manage resistance and limit herbicide use, while making good use of the unique powers of herbicides? |
| What are the most cost-effective new technologies? |
| What are the most cost-effective ways to control invasive species including plants, pests and pathogens? |
| Invasive species are eventually taking over. How do we deal with pest incursions? |
| Is it desirable and feasible to use zero-tolerance targets in our weed management programs? In which specific situations may this target be applied? |
| Up to now weed management has been conducted primarily at the field level with a time horizon of a few months (the current season). What specific improvements can be obtained by using other spatial scales (landscape, patch, plant) and time horizons (1-year period, 3- or 5-year rotations)? |
| Should weed research focus on delivering tailor-made solutions to the farmers or in establishing broad principles that can be applied to a variety of situations? |
| Is weed research becoming disengaged from practical agriculture? |
| Is it realistic to devise very complex weed management systems fitted to a specific situation or it would be more practical to focus on simple, widely used solutions? |
| What are the policies and technical lessons from the agriculturally developed world to the new agricultural developing world related to crop management and weed control? Is it just restrictions or should there be anything else? |
| Can GM approaches be used to modify crop traits in order to increase crop “resistance” to weed interference? |
| How can crops be manipulated for better weed management or site-specific weed management? |
| We all want to have herbicide free agricultural system - is it possible? |
| What is the value (both ecologically and economically) of control of different life history stages? |
| What factors do managers consider most important when choosing what and how to manage weeds / invasive plants? |
| When controlling agricultural pests and weeds through biological control are we overestimating the impact of surrounding semi-natural and natural habitats as reservoirs for our control agents? |
| When looking into the importance of reservoir habitats for control agents are we making a mistake when transferring any knowledge gained in temperate systems into subtropical and tropical ones? |
| How does competition influence the efficacy of herbicides? |
| What is hampering the adoption of integrated weed management strategies? What are farmers trying to tell us? |
| Is achieving completely weed free crop fields a valid aim of ‘modern’ weed science? |
| Is there really anything we can do to stop (or slow) the evolution of resistance to herbicides? |
| How can weed scientists contribute to the development of agricultural systems that are both highly productive and highly effective in safeguarding environmental quality and human health? |
| How can farming systems be designed for greater resilience with regard to weeds? |
| Do we need to put more research effort into the development of new Ecological Weed Management strategies or should we focus on what is hindering adoption and improve our communication skills about the outcomes of our research? |
| Weed problems are embedded in interactions across different levels. How do we account for interactions at plant, plot, farm, community, subnational, regional and national levels? |
| What next for research after IWM?  Do we just need more copy-cat, case study research on particular weeds in particular systems?  Where is innovation in management going to come from? |
| What would it take to get (most) farmers to change attitudes towards weeds/herbicides and adopt true IWM rather than integrated herbicide management?  Or are the economics/social/psychological issues such that they never do what “scientists” think is sensible? |
| Can we predict and manage for off-target effects of invasive plant management? |
| Impacts of invasive species may not always be reversible by removal, what additional management actions may be necessary? |
| The transgene facilitated herbicide treadmill is ramping up around the world, what meaningful steps can be taken to integrate weed management? |
| Do improved biosecurity practices reduce plant invasions? How do we provide evidence? |
| Can ecological weed management ever deliver the (short-term and short-sighted) 'success' of chemical weed management? Do we need to move the goalposts? |
| **Category: Socio-economics and policy** |
| How can weed ecologists best engage with civil society, government and private enterprise to organize multi-stakeholder efforts to manage plant invasion problems? |
| Can we devise a system of governance for new, potentially invasive crops (e.g., oilseed cultivars of field pennycress) that manages risks without “stifling innovation”? |
| Do policy makers take appropriate action in the face of new incursions? |
| How do we frame research questions in order to influence policy decisions? |
| How do political/economic changes affect weed invasion? Can it be predicted or prevented? |
| How can we help land managers and decision makers to access high quality information on weed prevention, identification and control? |
| How can integrated socio-ecological systems analysis be undertaken to better understand weed dispersal and management? |
| How does weed dispersal and management relate to characteristics of the associated social systems? |
| How does knowledge about weed management get shared/transformed across time and space? |
| Can weed ecology and social science theories be used to challenge and inform one another? |
| How can we work with social scientists to best co-ordinate weed prevention and control efforts amongst multiple land owners, land users and agencies? |
| How can farmer behaviour be best influenced to improve sustainability of weed management? |
| How can weed scientists contribute to the development of agricultural systems that better reward farm labour and management time? |
| **Category: Weed science as a discipline** |
| How can our research community avoid falling in the gap between ‘applied’ and ‘basic, hypothesis-driven’ research funding programs? |
| How can we attract excellent scholars into the field, and improve the training of current and future colleagues to include appropriate advanced, cutting edge computational genomics theory and tools? |
| Are colleagues and students learning enough basic science to be innovative? On the flip side, are students entering University as undergraduates or beginning graduate studies turning away from weedy and invasive species biology/genetics/etc.? If so, why? |
| How do we connect fundamental and applied research in weed research? |
| Weed problems have biophysical, technological, socio-cultural, economic, institutional and political dimensions. How do we integrate across these disciplines? |
| How do we foster approaches that focus on structural transformations to enhance the overall weed system’s capacity to innovate? |
| How do we widen the scope, relevance, application and attractiveness of research in weed ecology and management? |
| How do we enhance the importance of weeds (and weed science) in agricultural systems in comparison to other pests (phytophagus, fungi diseases, etc.)? |
| Would it to answer research questions (or some of them) if every researcher would put her/his data online, open for use to anyone interested? |
| How do we free our discipline from the neo-liberal agenda (private sector influence on issues of public interest)? |
| A noticeable narrowing in content has occurred (in North America at least) within the "Weed Science" community over the past decade, how do we move to broaden that scope? |
